# Preoperative insulin resistance reduces complications after hip replacement surgery in non-diabetic patients

**DOI:** 10.1186/1471-2253-13-39

**Published:** 2013-11-04

**Authors:** Robert G Hahn, Stefan Ljunggren

**Affiliations:** 1 Research Unit, Södertälje Hospital, Södertälje, Sweden; 2 Department of Anesthesiology, Faculty of Health Sciences, Linköping University, Linköping, Sweden; 3 Karolinska Institutet, Department of Clinical Science and Education, Södersjukhuset, Stockholm, Sweden

**Keywords:** Insulin resistance, Complication, Arthroplasty, Hip replacement

## Abstract

**Background:**

Insulin resistance negatively affects the outcome of surgery in patients with type 2 diabetes. This association is often believed to be present in other patient populations as well, but studies are lacking on the influence of preoperative insulin resistance on the clinical course of surgery in non-diabetic patients.

**Methods:**

Sixty non-diabetic patients with a mean age of 68 years underwent a 75-min intravenous glucose tolerance test (IVGTT) one day before and after elective hip replacement surgery. Patients were regarded to be either insulin resistant (< median insulin sensitivity) or not (> median insulin sensitivity). Hypotensive events occurring in the postoperative care unit and complications in the orthopedic ward were recorded. Fatigue and well-being were assessed via questionnaires.

**Results:**

A total of 52 patients were included in the final analysis. Insulin resistance before surgery was associated with a lower risk of arterial hypotension in the postoperative care unit (systolic pressure < 80 mmHg; *P* < 0.05) and with fewer complications in the orthopedic ward (mean 1.9 *versus* 1.2 per operation, *P* < 0.01), particularly with respect to nausea/vomiting (*P* < 0.04) and arterial hypotension (*P* < 0.05). Fewer of these patients had more than one complication (23% *versus* 58%, *P* < 0.001), while no statistical link between preoperative insulin resistance and fatigue or well-being was evident. Insulin resistance, when measured one day postoperatively, did not correlate with the number of complications.

**Conclusions:**

Preoperative insulin resistance offers some benefit in the postoperative period and early convalescence in non-diabetic patients who undergo hip replacement surgery.

## Background

Insulin resistance is an important aspect of glucose metabolism and implies that only a small amount of glucose is cleared from the extracellular fluid in response to any given insulin secretion [1]. If insulin resistance is pronounced, as in type 2 diabetes, even a high level of insulin secretion is insufficient to maintain the plasma glucose concentration within the normal range.

Insulin resistance may show a ten-fold variation even in non-diabetic subjects [2], but the clinical significance of this variation is little appreciated. Diabetes is known to increase the risk of postoperative complications [3], while the possible influence of insulin resistance on the surgical outcome in non-diabetic patients has not been studied.

The present report is an exploratory analysis to determine whether insulin resistance influences the incidence of postoperative complications and well-being after elective hip replacement surgery.

The hypothesis is that insulin resistance increases the number of complications in non-diabetic patients, just as it does in those with diabetes. To study this issue, we compared complications and well-being in non-diabetic patients depending on their degree of insulin resistance, as determined by a short Intravenous Glucose Tolerance Test (IVGTT) [2,4,5].

## Methods

The participants were 60 patients aged between 44 and 89 years (mean, 69 years) who underwent elective total hip replacement under spinal anesthesia between May 2008 and September 2009. All patients gave their informed consent to participate in the study. Exclusion criteria included diabetes and other endocrinological diseases.

The protocol was approved by the Regional Ethics Committee of Stockholm (Ref. 2007/1628-31/4) as part of a clinical trial comparing the effects of preoperative fasting *versus* the ingestion of water with and without carbohydrates on postoperative outcomes (ClinicalTrials.gov NCT01211184). These treatments were found to have virtually identical outcomes [4], so composite data are used in the current presentation.

Total hip replacement surgery was performed under spinal anesthesia with 2.5–4 ml bupivacaine at 5 mg/ml (AstraZeneca, Södertälje, Sweden). Routine fluid therapy during the operations consisted of 1,000 ml of acetated Ringer’s solution and 500 ml of hydroxyethyl starch at 130/0.4 (Voluven, Fresenius Kabi, Bad Homburg, Germany). However, two patients received no starch, and four were given 1,000 ml of the starch. Blood loss was taken as the sum of the visually estimated amounts present on swabs and dressings and the measured volume found in suction bottles.

For postoperative pain relief, 22 patients received an epidural catheter with a continuous infusion of levobupivacaine at 4–7 mg/h and sufentanil at 3–6 μg/h. Twenty-five patients were given a wound catheter instead, in which 150 mg of ropivacaine and 30 mg of ketorolac were injected at the end of surgery and during the first postoperative morning. All patients were also given oral acetaminophen, and oral oxycodone served as rescue pain reliever.

### Insulin resistance

Insulin resistance was calculated from a 75-min, seven-sample IVGTT performed in the fasting state on the day before the surgery and also on the morning of the first postoperative day. Before the preoperative IVGTT, the patients had fasted for 4 hours, while before the second, they had fasted overnight.

The insulin resistance was obtained by dividing the slope of the mono-exponential elimination curve for glucose by the area under the curve for plasma insulin above baseline (AUC_ins_) during the IVGTT. This approach recently showed a good correlation with the euglycemic hyperinsulinemic glucose clamp in volunteers [2,5], as well as in diabetic and postoperative patients [5].

The mono-exponential elimination curve obtained from a short IVGTT might be formed by the ratio between the volume of distribution (*V*_d_) and the clearance (*CL*) of the injected glucose. Based on a previous study in volunteers [2], the relationship between the rate of glucose uptake (M_bw_) given by the clamp method and *V*_d_, *CL* and AUC_ins_ given by the IVGTT after an intravenous injection of 0.3 g/kg of glucose can be expressed by the following regression equation:

$$M_{\text{bw}}=45.4\;\times {\;}^{10}\log\;\left[\frac{\text{CL}\cdot 10^{6}}{V_{d}\cdot \text{AU}C_{\text{ins}}}\right]$$

The glucose uptake given by the clamp, which is usually denoted M, was corrected for body weight (M_bw_) to account for the fact that a heavy person is likely to take up relatively more glucose at maximum insulin stimulation. Moreover, the bolus dose of glucose given as part of the IVGTT was adjusted for body weight. The reader should note that a *low* M_bw_ implies insulin resistance.

In the previous study in volunteers, the hyperinsulinemic glucose clamp yielded values of M_bw_ ranging from 9 to 62 μmol/min/kg [2] which the equation cited above could predict from an IVGTT with 25–75^th^ percentiles of −10% and +16% and a linearity (correlation coefficient) of 0.80.

An IVGTT of 0.3 g/kg instantly expands the plasma volume by 10% [6], which might cause cardiovascular strain in the elderly. Therefore, the dose of glucose was reduced to 0.2 g/kg, and the intravenous injection time was increased from 1 to 4 min, as suggested previously [6]. The glucose was administered as a 30% solution, and the plasma glucose and insulin concentrations were measured at baseline and at 10, 20, 30, 45, 60, and 75 min.

The plasma glucose concentration was analyzed on a Modular P (Roche Diagnostics, Tokyo, Japan), and plasma insulin was analyzed on a Roche-Hitachi Modular E170 (Hitachi, Tokyo, Japan). *CL* and *V*_d_ were estimated via a Gauss-Newton least-squares regression routine that was applied to a one-compartment kinetic model, which had been entered into the computer program Matlab 4.2 (Math Works Inc., Natick, MA) as described elsewhere [2,4,7].

The IVGTT-derived data on insulin resistance (M_bw_) were dichotomized, and patients were characterized as being insulin resistant or not, depending on whether M_bw_ was below or above the median for the cohort.

### Complications

The medical records from the Postoperative Care Unit (PACU), where the patient spent the first 3–4 hours after the surgery, were used to let a blinded investigator count the number of early hypotensive events, which were defined as a systolic arterial pressure < 80 mmHg.

On the morning of the second postoperative day (48 hours after surgery), a research nurse used a checklist of 18 complications based on the previously published schemes by Bennett-Guerrero *et al.*[8] and Brandstrup *et al.*[9] to register adverse events occurring in the orthopedic ward after the patient had left the PACU. The registered complications summarized the problems that appeared during the postoperative care period, excluding the hours spent in the PACU when residual effects of anesthetic drugs could have affected the clinical picture.

The listed complications were: need for ventilation or oxygen, fever (>38°C), pain requiring parenteral rescue opioid treatment, nausea and vomiting, hypotension (requiring pharmacological therapy or fluid therapy), cardiac arrhythmias, food intolerance, neurological problems (confusion, etc.), headache, wound complications, blood transfusion, kidney problems (oliguria < 500 ml per day or rise in serum creatinine of >30%), sepsis, pneumonia, pulmonary edema, myocardial infarction, stroke, and cystitis (urgency and pain on voiding). Blood transfusion was later removed from the list because the authors considered it to be an expected result of the surgery.

### Fatigue and well-being

As surgery and insulin resistance may influence well-being [5,10,11], the patients answered questionnaires about fatigue and quality of life in the afternoon on the day before the surgery and then again two weeks after the operation had been performed.

The degree of fatigue was assessed using the Fatigue scale Questionnaire (FQ), which consists of 14 questions covering both the physical and mental aspects of fatigue [12,13]. A “yes” answer to a question about the presence of fatigue is graded as a 1, and a “no” answer is graded as a 2. Uncertain answers are graded as 1.5.

The overall quality of life was assessed via the Health Index (HI) questionnaire [14,15]. The patient responds to ten questions about “energy,” temper, mood, fatigue, loneliness, sleep, vertigo, bowel function, pain, mobility, and overall health during the past week. Answers can be given on four levels: 1 = very poor; 2 = rather poor; 3 = rather good; and 4 = very good. Hence, the highest HI score is 40, and the lowest is 10.

### Statistics

Data showing normal distributions were presented as the means ± standard deviations. Differences between groups were evaluated via one-way ANOVA. Data having a skewed distribution were reported as the median (25^th^–75^th^ percentiles), and the differences were studied via the Mann–Whitney U test. Binary data were evaluated via contingency table analysis, and the relationships between binary outcomes and other parameters were assessed by logistic regression analysis. The statistical software was StatView SE + Graphics for Mac (Abacus Concepts Inc., Berkeley, CA, USA) but the logistic regression analysis was performed by SPSS version 20 (Statistical Package for Social Sciences; IBM, Chicago, 2011). No formal power analysis was performed because of the *post hoc* nature of this report [4]. *P* < 0.05 was considered statistically significant.

## Results

### Basic data

Three patients dropped out of the study, and the occasional hemolysis of insulin samples further reduced the number of evaluable patients to 52.

Before the surgery, the highest plasma glucose concentration was 6.5 mmol/L (diabetes is defined as >7.0 mmol/L). The median insulin resistance (M_bw_) for the cohort was 31 (19–40) μmol/min/kg. Thus, the 26 patients with M_bw_ of 31 μmol/min/kg and lower were denoted “insulin resistant” in the following presentation.

Table 1 shows demographic and blood chemistry data in the presence or absence of insulin resistance.

**Table 1:** Demographics and blood chemistry of patients undergoing elective hip replacement surgery

**Parameter**	Insulin resistance* (n = 26)	**No insulin resistance (n = 26)**	**Univariate statistics**
Age (years)	66 ± 10	70 ± 9	NS
Body weight (kg)	90 ± 16	75 ± 11	*P* < 0.001
Female / male (N)	15 / 11	20 / 6	NS
Placebo / water / nutrition (N)	10 / 8 / 8	10 / 7 / 9	NS
Preoperative blood chemistry			
Plasma glucose (mmol/l)	5.0 ± 0.4	5.1 ± 0.7	NS
Plasma insulin (pmol/l)	61 (33–99)	29 (24–43)	*P* < 0.003
Insulin sensitivity (μmol/min/kg)	19 (9–24)	41 (36–46)	*P* < 0.001
Blood hemoglobin (g/L)	137 ± 10	130 ± 12	*P* < 0.05
Postoperative blood chemistry**			
Plasma glucose (mmol/l)	5.9 ± 1.2	5.7 ± 1.0	NS
Plasma insulin (pmol/l)	57 (42–73)	26 (18–50)	*P* < 0.002
Insulin sensitivity (μmol/min/kg)	11 (2–18)	21 (13–34)	*P* < 0.003
Blood hemoglobin (g/L)	103 ± 16	102 ± 13	NS

*Insulin resistance implied that the insulin sensitivity was below the median for the cohort.

**Postoperative samples were collected in the morning on the first day after the surgery.

### Hypotension in the PACU

**Figure 1:**
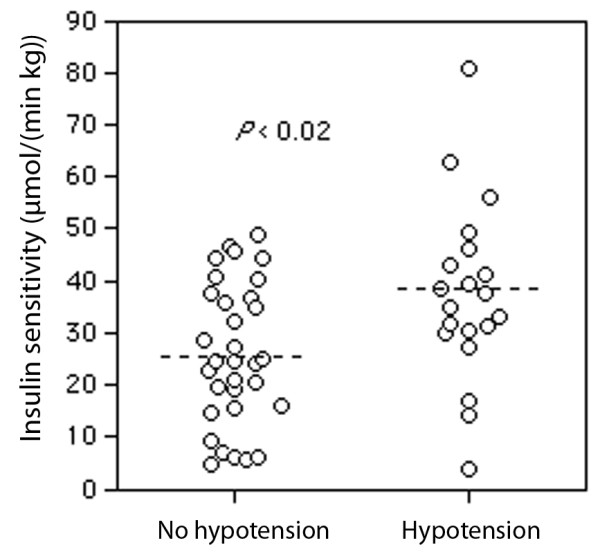
**The incidence of arterial hypotension in the postoperative ward (systolic blood pressure < 80 mmHg).** Low insulin sensitivity implies high insulin resistance. Each point represents one patient. The dashed lines indicate median values.

Patients with insulin resistance before surgery were more prone to develop arterial hypotension in the PACU (systolic pressure < 80 mmHg; Figure 1, Table 2).

**Table 2:** Systolic arterial pressure, surgical data, and well-being of patients undergoing hip replacement

**Parameter**	**Insulin resistance (n = 26)**	**No insulin resistance (n = 26)**	**Univariate statistics**
Systolic arterial pressure (mmHg)			
Before spinal anesthesia	153 ± 20	152 ± 17	NS
After induction	103 ± 24	98 ± 26	NS
Mean during surgery	113 ± 24	111 ± 11	NS
Mean in postoperative care unit	106 ± 11	100 ± 13	NS
Events below 80 mmHg (N)	6	14	*P* < 0.05
Operating time (min)	104 ± 22	109 ± 25	NS
Blood loss (ml)	400 (300–500)	400 (300–650)	NS
Erythrocyte transfusions (N patients)	4	8	NS
Postoperative epidural anesthesia (N)	16	16	NS
Length of hospital stay (days)	5.7 ± 1.4	6.0 ± 1.3	NS
Fatigue scale (FQ) questionnaire (score)			
Before surgery	23.3 ± 3.9	23.2 ± 3.8	NS
2 weeks after surgery	25.4 ± 2.2	25.1 ± 2.3	NS
Health index (HI) questionnaire (score)			
Before surgery	29 (23–32)	29 (26–31)	NS
2 weeks after surgery	31 (30–33)	31 (29–35)	NS

NS = not significant.

Logistic regression analysis showed that the occurrence of hypotension in the PACU correlated independently with the insulin sensitivity before surgery (*P* < 0.011) but not with the use of epidural anesthesia, blood loss, or body weight.

### Complications in the orthopedic ward

**Figure 2:**
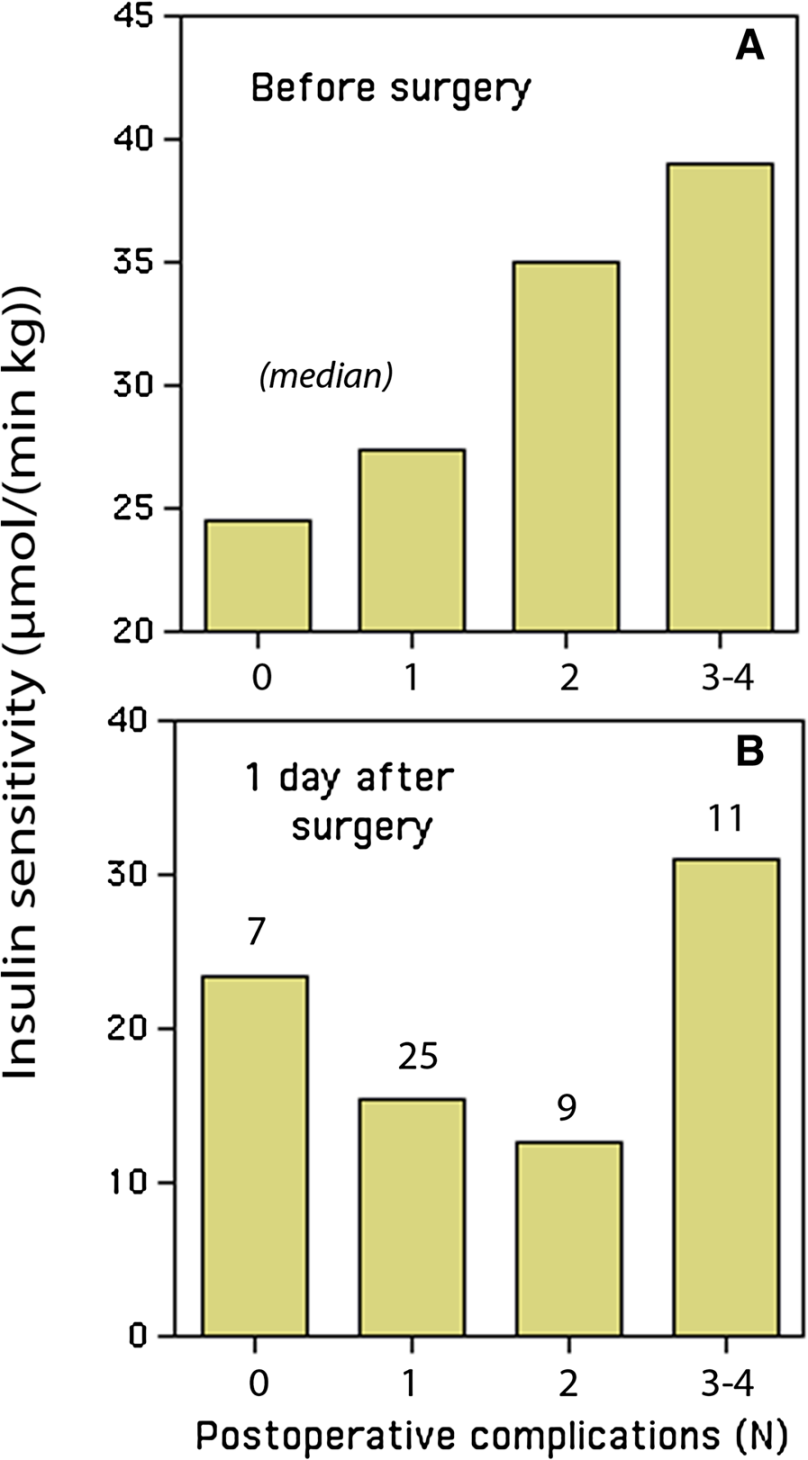
**Insulin sensitivity before (A) and after (B) elective hip replacement surgery.** Low insulin sensitivity = insulin resistance. A research nurse recorded the number of complications 2 days postoperatively. The numbers shown on top of the bars in B denote the number of patients.

Patients with insulin resistance before surgery had a lower incidence of complications in the orthopedic ward, as recorded during the follow-up 2 days after the surgery (mean 1.2 *versus* 1.9 per operation, *P* < 0.01; Figure 2). They were also less prone to have more than one complication (23% *versus* 58%, *P* < 0.001).

The lower complication rate was primarily due to less frequent problems with nausea and vomiting (19% *versus* 46%, *P* < 0.04) and arterial hypotension (23% *versus* 50%, *P* < 0.05; Table 3).

**Table 3:** Complications recorded in the orthopedic ward 2 days after surgery

**Parameter**	**Insulin resistance (n = 26)**	**No insulin resistance (n = 26)**	**Contingency table analysis**
Need for ventilation or oxygen	2	2	
Fever (>38^o^ C)	2	4	
Parenteral opioids	14	13	
Nausea and/or vomiting	5	12	*P < 0.04*
Hypotension	6	13	*P < 0.04*
Food intolerance	1	4	
Wound complication	1	0	
Kidney problems	1	0	
Headache	0	2	
Sepsis, pneumonia, cardiac arrhythmias, myocardial infarction, stroke, or cystitis	0	0	
Total	32	50	P < 0.01 *

* Analysis was performed by the Mann–Whitney U test.

Logistic regression analysis showed that hypotension in the orthopedic ward became more common with less pronounced insulin resistance before the surgery (*P* < 0.04) and also with increased use of epidural anesthesia (*P* < 0.02). In patients with insulin resistance, the risk of developing late hypotension was 9% (no epidural) and 30% (with epidural), while the corresponding figures for those without insulin resistance were 31% and 80%, respectively.

### Length of hospital stay and well-being

No overall correlation was noted between preoperative insulin resistance and the length of hospital stay. However, the patients with the most pronounced insulin resistance (M_bw_ < 25^th^ quartile) had longer hospital stays compared to the others (6.9 days *versus* 5.6 days; *P* < 0.01).

The FQ and HI scores before or 2 weeks after surgery were virtually identical among those with and without insulin resistance prior to surgery (Table 2).

### Postoperative insulin resistance

Those with insulin resistance measured in the first morning *after* surgery (< median 18.0 μmol/min/kg) reported greater improvement in terms of the FQ score 2 weeks postoperatively than the other patients did (change +1 *vs.* +3 points; *P* < 0.01). This difference was not statistically related to hypotensive events or erythrocyte transfusions. In contrast, postoperative insulin resistance did not correlate with the HI scores.

## Discussion

The report finds preoperative insulin resistance to be relevant to the clinical course of elective hip replacement surgery in non-diabetic patients. Insulin resistance was associated with a decreased risk of arterial hypotension developing in the PACU. Insulin resistance was also followed by a lower number of complications in the orthopedic ward, to which reductions in nausea, vomiting, and further hypotensive events primarily contributed. The more stable hemodynamics probably explains why these patients tended to receive erythrocytes less often than the others did, although they did not bleed less. In contrast, insulin resistance before surgery had limited bearing on the length of hospital stay, and no statistical relationship was found with pre- or postoperative fatigue or quality of life. Hence, the study hypothesis was not only refuted, but the findings, in fact, were opposite to the assumed ones.

Except for a glucose-sparing effect, the insulin resistance associated with physiological stress and trauma is poorly understood from a phylogenetic point of view. The present results are of interest in this context because they suggest that preoperative insulin resistance was of some benefit to our patients by improving their ability to cope with surgery. No negative consequences could be discerned here, although this scenario will differ when insulin resistance becomes sufficiently severe to cause type 2 diabetes.

Interpretation of the results is somewhat complicated by the fact that insulin resistance usually occurs concomitantly with other components of the “metabolic syndrome.” In the present study, patients with insulin resistance had higher body weights and plasma insulin levels at baseline than the others did, while the systolic arterial pressure was virtually identical in the two groups (Table 1). As expected, insulin resistance was also aggravated by the surgery. At the time when complications began to arise at the orthopedic ward, the insulin sensitivity was approximately half as high as it had been before the operations (Table 2), which is in accordance with previous results obtained via the glucose clamp method [16]. However, a correlation of insulin resistance with complications was only apparent when it was measured prior to the surgery (Figure 2).

Insulin resistance is usually believed to have negative implications. Any aggravation of the resistance during surgery should therefore be prevented, if possible, and this view has hitherto been widely accepted. Control of trauma-induced insulin resistance can be achieved via intravenous infusions of glucose and insulin [17,18], while a more practical prevention is to ingest a carbohydrate drink. The ability of one specific drink (Preop, Nutricia, Wiltshire, UK) to prevent surgery-induced insulin resistance has received some support from two small studies with high-quality assessments of glucose metabolism.

The first study comprised 15 patients who underwent elective hip replacement surgery, but the study group had markedly greater insulin resistance than the placebo group did before the surgery [16]. In the present work, patients with insulin resistance at baseline usually had a smaller absolute surgery-induced decrease in insulin sensitivity; the only exception being those with very many complications (Figure 2).

The second study included 13 abdominal surgery patients and needed to incorporate the operating time as a covariate in order to show efficacy [19]. The carbohydrate drink significantly reduced nausea and vomiting in a large group of patients undergoing laparoscopic cholecystectomy [10], although these preventive effects have not been consistent [11,20]. Others have claimed additional soft benefits, such as less thirst while awaiting surgery. However, none of these studies have evaluated the effect of preoperative insulin resistance *per se* on postoperative complications and well-being.

Limitations include that surgical hemorrhage was assessed clinically, i.e. as the summary of the measured volume in suction bottles and visually estimated amounts present on swabs and dressings. With this approach blood loss did not differ depending on the degree insulin resistance, but it is possible that a difference had been disclosed if the lost blood had been had washed out from the swabs by saline and the amount measured by a photometer.

The choice of cutoff point for arterial hypotension can be debated. During the induction of anesthesia, an acute reduction of the systolic pressure by 25–30% or more is often regarded as representing hypotension [21]. However, our criterion is justified by the fact that the postoperative hypotension was subacute [22]; most of the total decrease in arterial pressure had already occurred during anesthesia and surgery (Table 2). Moreover, systolic pressures below 80 mmHg attenuate the autoregulation of the renal blood flow and the glomerular filtration rate, and are frequently accompanied by near-fainting.

Another limitation is that insulin sensitivity was measured via IVGTT and not via the glucose clamp, although the former is claimed to be a fully acceptable alternative [1]. The clamp was considered unsuitable for this study because it induces an extreme physiological situation for 3–4 hours, which could have modified the incidence of surgical complications. One should also be aware that the results are based on exploratory analyses from a clinical trial [4]. Although the clinical implication here is that efforts to reduce insulin resistance prior to surgery are hardly worthwhile, this view needs to be challenged in a prospective study.

## Conclusion

Preoperative insulin resistance in non-diabetic patients was associated with a lower incidence of complications after elective hip replacement surgery performed under spinal anesthesia.

## Abbreviations

AUC: Area under the curve; CL: Clearance; IVGTT: Intravenous glucose tolerance test; r: Correlation coefficient; Vd: Volume of distribution; T½: Half-life; Mbw: Glucose uptake during the last 30 minutes of an euglycemic hyperinsulinemic glucose clamp.

## Competing interests

The authors declare that they have no competing interest.

## Authors’ contributions

SL recruited and informed the patients, was responsible for references and co-wrote the manuscript. RGH planned the study, wrote appropriate applications, and had the main responsibility for the content of the manuscript. Both authors read and approved the final manuscript.

## Authors’ information

Stefan Ljunggren is an MD and specialist in orthopedics. He is currently a PhD Student. Robert G. Hahn is an MD is professor of anesthesiology and intensive care.

## Pre-publication history

The pre-publication history for this paper can be accessed here:

http://www.biomedcentral.com/1471-2253/13/39/prepub
